# Generalized Fast Discharges Along the Genetic Generalized Epilepsy Spectrum: Clinical and Prognostic Significance

**DOI:** 10.3389/fneur.2022.844674

**Published:** 2022-03-10

**Authors:** Emanuele Cerulli Irelli, Francesca Antonietta Barone, Luisa Mari, Alessandra Morano, Biagio Orlando, Enrico Michele Salamone, Angela Marchi, Martina Fanella, Jinane Fattouch, Fabio Placidi, Anna Teresa Giallonardo, Francesca Izzi, Carlo Di Bonaventura

**Affiliations:** ^1^Epilepsy Unit, Department of Human Neurosciences, Policlinico “Umberto I”, Sapienza University, Rome, Italy; ^2^Epilepsy Center, Neurology Unit, University Hospital of Rome Tor Vergata, Rome, Italy

**Keywords:** generalized paroxysmal fast activity (GPFA), generalized polyspike train (GPT), idiopathic generalized epilepsy (IGE), prognosis, prolonged ambulatory EEG (paEEG), antiseizure medication (ASM), classification, 24-hour EEG

## Abstract

**Objective:**

To investigate the electroclinical characteristics and the prognostic impact of generalized fast discharges in a large cohort of genetic generalized epilepsy (GGE) patients studied with 24-h prolonged ambulatory electroencephalography (paEEG).

**Methods:**

This retrospective multicenter cohort study included 202 GGE patients. The occurrence of generalized paroxysmal fast activity (GPFA) and generalized polyspike train (GPT) was reviewed. GGE patients were classified as having idiopathic generalized epilepsy (IGE) or another GGE syndrome (namely perioral myoclonia with absences, eyelid myoclonia with absences, epilepsy with myoclonic absences, generalized epilepsy with febrile seizures plus, or GGE without a specific epilepsy syndrome) according to recent classification proposals.

**Results:**

GPFA/GPT was found in overall 25 (12.4%) patients, though it was significantly less frequent in IGE compared with other GGE syndromes (9.3 vs. 25%, *p* = 0.007). GPFA/GPT was found independently of seizure type experienced during history, the presence of mild intellectual disability/borderline intellectual functioning, or EEG features. At multivariable analysis, GPFA/GPT was significantly associated with drug resistance (*p* = 0.04) and with a higher number of antiseizure medications (ASMs) at the time of paEEG (*p* < 0.001) and at the last medical observation (*p* < 0.001). Similarly, GPFA/GPT, frequent/abundant generalized spike-wave discharges during sleep, and a higher number of seizure types during history were the only factors independently associated with a lower chance of achieving 2-year seizure remission at the last medical observation. Additionally, a greater number of GPFA/GPT discharges significantly discriminated between patients who achieved 2-year seizure remission at the last medical observation and those who did not (area under the curve = 0.77, 95% confidence interval 0.57–0.97, *p* = 0.02)

**Conclusion:**

We found that generalized fast discharges were more common than expected in GGE patients when considering the entire GGE spectrum. In addition, our study highlighted that GPFA/GPT could be found along the entire GGE continuum, though their occurrence was more common in less benign GGE syndromes. Finally, we confirmed that GPFA/GPT was associated with difficult-to-treat GGE, as evidenced by the multivariable analysis and the higher ASM load during history.

## Introduction

Generalized paroxysmal fast activity (GPFA) has always been considered one of the electroencephalography (EEG) hallmarks of Lennox-Gastaut syndrome (LGS). GPFA typically occurs during sleep and may be associated with the occurrence of tonic seizures (1).

GPFA was later described in genetic generalized epilepsy (GGE) patients exhibiting intermediate phenotypes between classical idiopathic generalized epilepsy (IGE) and epileptic/developmental encephalopathy, with higher rates of poor seizure control and borderline intellectual functioning (2, 3). However, different authors have recently described GPFA in classical IGE syndromes with favorable seizure and neuropsychiatric outcomes, raising questions regarding the real prognostic and electroclinical significance of this EEG pattern along the GGE spectrum (4–6). In addition to GPFA, a newly described EEG pattern, defined as generalized polyspike train (GPT), has been reported in GGE patients (7). GPT is characterized by a shorter discharge duration and has been considered a promising biomarker of drug resistance in GGE patients (7, 8).

Despite the potential relevance of these EEG fast discharges in GGE outcome assessment, few data from 24-h prolonged ambulatory EEG (paEEG) recordings are currently available (7, 8). In fact, paEEG may represent an invaluable tool in order to capture quality sleep, when GPFA/GPT are more likely to occur (7), and to properly assess the occurrence and the characteristics of these peculiar EEG patterns.

In addition, the majority of previous studies focusing on GPFA have been limited to case series describing the electroclinical features of a subgroup of patients showing these EEG patterns, and no authors have specifically investigated the electroclinical differences between patients with and without GPFA/GPT in a large cohort of GGE patients.

With this background, we aimed to investigate generalized fast discharges in a large cohort of GGE patients studied with paEEG in order to clarify their prevalence and identify the electroclinical characteristics and epilepsy syndromes associated with these EEG patterns along the GGE spectrum. Finally, we aimed to explore the prognostic significance of GPFA/GPT by analyzing their association with different seizure outcome measures.

## Methods

### Study Participants, Setting, and Eligibility Criteria

The study was conducted according to Strengthening the Reporting of Observational Studies in Epidemiology (STROBE) guidelines as a retrospective multicenter cohort study and was approved by the local ethics committees. Data from patients followed at Policlinico Umberto I (Rome, Italy) and Policlinico Tor Vergata (Rome, Italy) adult epilepsy outpatient clinics from 2008 to 2020 were retrospectively reviewed.

Patients were enrolled according to the following inclusion criteria: (1) diagnosis of GGE according to recently proposed criteria; (9) (2) availability of at least one 24 h ambulatory EEG and one standard video EEG; (3) availability of complete clinical documentation in order to adequately review electroclinical features and seizure outcome information; and (4) follow-up duration of at least 5 years from epilepsy onset to the last medical observation and at least 24 months from paEEG recording to the last medical observation. Patients with cognitive deficits other than borderline intellectual functioning and/or mild intellectual disability (ID) were excluded in order to minimize the risk of including patients with underlying epileptic/developmental encephalopathies.

GGE syndromes were classified by two trained epileptologists (ECI, ATG), according to the latest International League Against Epilepsy (ILAE) classification proposals (9, 10). In case of discrepancies between ECI and ATG, consensus was reached by discussion with a third epileptologist (AM). According to the latest proposals, the following GGE syndromes were identified: (1) four IGE syndromes, namely childhood absence epilepsy (CAE), juvenile absence epilepsy (JAE), juvenile myoclonic epilepsy (JME), and IGE with generalized tonic-clonic seizure alone (GTCSA); (2) GGE syndromes other than IGE, namely eyelid myoclonia with absences (EMA), epilepsy with myoclonic absences, myoclonic epilepsy in infancy, myoclonic-atonic epilepsy, and generalized epilepsy with febrile seizures plus (GEFS+) (11–13).

Perioral myoclonia with absences was also considered in the study to achieve a more accurate clinical characterization of the patient population (14), although not yet included in the classification proposal. GGE patients not fulfilling the criteria for any recognizable epilepsy syndrome were considered as having GGE without a specific epilepsy syndrome (hereinafter referred to as “undefined GGE”) (9). The diagnostic criteria used to classify patients into different GGE syndromes are thoroughly reported in Supplementary Material.

### Clinical Data Collection and paEEG Assessment

Clinical charts were reviewed for demographic data, family history of epilepsy in first- or second-degree relatives, personal or family history of febrile seizures (FS), age at epilepsy onset, follow-up duration, psychiatric comorbidities, presence of mild ID and/or borderline intellectual functioning (as evidenced by a standardized neuropsychological assessment), seizure types and frequency, MRI findings (when available), and drug regimen changes.

PaEEG recordings were reviewed page-by-page with 20-s pages on a longitudinal bipolar montage with a 1.6–70 Hz bandwidth. The following features and abnormalities were evaluated: (1) generalized spike-wave discharges (SWDs) and polyspike-wave discharges (PWDs), defined as one or more spikes lasting from 20 to 80 ms followed by a surface negative wave, with their relative frequencies; (2) the presence of focal epileptiform abnormalities, defined as focal discharges confined to a single lobe; (3) asymmetry of SWDs or PWDs, both in terms of onset (exceeding 200 ms) and amplitude (more than 30% of maximum discharge amplitude); (4) presence of focal slow waves; and (5) presence of GPFA and/or GPT. In case of GPFA/GPT occurrence, the following characteristics were analyzed in each patient: number of discharges during paEEG, maximum amplitude, duration, possible relation to SWDs, and the EEG epoch of occurrence (namely wakefulness, NREM sleep, REM sleep). The prevalence of SWDs and/or PWDs during paEEG was categorized according to ACNS criteria as follows: none, rare (<1/hour), occasional (≥1/hour but less than 1/minute), frequent (≥1/minute but less than 1 per 10 seconds), abundant (≥1 per 10 seconds, but not periodic) (15). The prevalence of PWDs/SWDs was independently estimated during wakefulness and during sleep in each patient.

GPFA was conventionally defined as a generalized discharge of rhythmic polyspikes in beta frequency with a duration of at least 1 s with frontal predominance (6), while GPT was defined according to the new definition proposed by Sun et al. as more than five generalized rhythmic polyspikes lasting less than 1 s (6).

Standard EEGs were reviewed in order to assess background activity characteristics and the occurrence of a photoparoxysmal response after intermittent photic stimulation.

Both standard EEGs and paEEGs were independently reviewed by two trained epileptologists (ECI, ATG) blinded to outcome measures. If a patient had multiple paEEG studies available for review, we chose to review the first paEEG study performed at each epilepsy outpatient clinic. The prevalence of SWDs and PWDs was manually scored, as well as the number and characteristics of GPT/GPFA discharges. EEG and clinical data were entered into a custom-made electronic database by the same epileptologists who reviewed the paEEGs (ECI, ATG).

### Clinical Outcomes

To study the relationship between GPFA/GPT and treatment response, different outcome measures were considered: (1) early remission, defined as the occurrence of 1+ years of seizure freedom within 2 years of epilepsy onset that persisted over the entire follow-up duration (16); (2) two-year seizure remission at the last medical observation; (3) seizure freedom during the 12 months prior to paEEG recording was also assessed, considering separately the occurrence of freedom from all seizure types and from specific seizure types (namely absences, myoclonic seizures, and GTCS); (4) drug resistance, defined as the failure of at least two established ASMs given in adequate dosages, either alone or in combination (17); (5) the number of ASMs at the moment of paEEG recording; and (6) the number of ASMs at the last medical observation.

### Statistical Analysis

Data were tested for normal distribution using the Shapiro–Wilk test and data visualization methods. Data were therefore presented as mean ± standard deviation (SD) or median [interquartile range (IQR)], as appropriate. Continuous data were compared across relevant groups using the Student *t-*test for normally distributed variables and the Mann–Whitney *U* test for non-normally distributed variables. Categorical variables were presented as counts (percentages) and compared using Fisher exact test. Group tests were two-sided with *p* < 0.05 considered statistically significant.

The receiver operating characteristic (ROC) curve was used to investigate the prognostic value of the number of GPFA/GPT discharges and the relative area under the curve (AUC), while the 95% confidence interval (CI) expressed the inherent ability of this EEG pattern to discriminate between patients with or without a 2-year seizure remission at the last medical observation.

Two different multivariable logistic regression models were elaborated to test the association between GPFA/GPT and: (1) the occurrence of 2-year seizure remission at the last medical observation; (2) drug resistance, as defined above. The following variables were included in the analysis: age at epilepsy onset (years), female sex, family history of epilepsy in 1^st^ and/or 2^nd^ degree relatives, presence of mild ID/borderline intellectual functioning, personal history of febrile seizures, photosensitivity, eye closure sensitivity, psychiatric comorbidities, number of seizure types ever experienced, GPFA/GPT occurrence during paEEG, focal epileptiform discharges observed during paEEG, prevalence of SWDs/PWDs during wakefulness, prevalence of SWDs/PWDs during sleep. Due to heterogeneous time intervals from epilepsy onset to paEEG recording and from paEEG recording to the last medical observation, the first model was adjusted for both the time from epilepsy onset to paEEG and the time from paEEG to the last medical observation, whereas the second model was adjusted for the time from epilepsy onset to the last medical observation. All independent variables showing a p value < 0.2 at univariable analysis were included in the multivariable model. Results were presented as odds ratios (ORs) with 95% CIs.

To confirm the possible association between GPFA/GPT and the number of ASMs used at different timepoints (namely the number of ASMs at the moment of paEEG and at the last medical observation), two multiple linear regression models were used to adjust the analysis for the same variables explained above. Only independent variables showing a *p* value < 0.2 at univariable analysis were included in multivariable models. The number of ASMs at different timepoints (namely the number of ASMs at the time of paEEG and at the last medical observation) was used as a dependent variable in each model.

IBM SPSS Statistics version 27 for Windows 10 (IBM Corp., Armonk, NY, USA) was used in the data analysis.

## Results

### General Characteristics of the Study Cohort

After identifying 601 GGE patients from both epilepsy outpatient clinics, 202 GGE patients were enrolled according to the inclusion criteria (Supplementary Figure 1). The median age of study participants at the last medical observation was 33 years (IQR 26–44.3) and 114 subjects (56.4%) were female. The median age at epilepsy onset was 13 years (IQR 8.8–17) and the median time from epilepsy onset to the last medical observation was 19 years (IQR 12–32.3). The median time from paEEG recording to the last medical observation was 7 years (IQR 4–12).

### Electroclinical Characteristics

A family history of epilepsy in a first- or second-degree relative was observed in 63 patients (31.2%), whereas a family history of FS in a first- or second-degree relative was observed in 16 (7.9%). A history of FS was observed in 21 patients (10.4%). Borderline intellectual functioning and/or mild ID were observed in 18 patients (8.9%), psychiatric comorbidities were observed in 26 patients (12.9%). Standard EEG [a treatment-naïve EEG was available for 47/202 patients (23.3%)] revealed a photoparoxysmal response in 54 patients (26.7%).

Regarding seizure types ever experienced during history, absences were observed in 98 patients (48.5%), GTCS in 186 (92.1%), myoclonic seizures in 77 (38.1%), eyelid myoclonia with/without absences in 24 (11.9%), myoclonic absences in 2 (1%), perioral myoclonia with absences in 2 (1%).

The most common epilepsy syndrome observed in our cohort was JME in 72 patients (35.6%), followed by GTCSA in 48 (23.8%), CAE in 23 (11.4%), JAE in 19 (9.4%), EMA in 15 (7.4%), undefined GGE in 15 (7.4%), GEFS+ in 6 (3%), perioral myoclonia with absences in 2 (1%), and epilepsy with myoclonic absences in 2 (1%).

The main electroclinical characteristics according to different GGE sub-syndromes are summarized in Table 1, whereas a detailed description of the four IGE syndromes is provided separately in Supplementary Table 1.

**Table 1:** Main electroclinical characteristics stratified by epilepsy syndrome.

	**IGE (162 pts)**	**EMA (15 pts)**	**Undefined GGE (15 pts)**	**GEFS+ (6 pts)**	**Myoclonic absences (2 pts)**	**Perioral myoclonia (2 pts)**
Female sex, *n* (%)	91 (56.17)	9 (60)	8 (53.33)	5 (83.33)	0	1 (50)
Age at epilepsy onset, years, mean (SD)	14 (7.5)	8 (3.2)	15.7 (7.7)	10.7 (8.7)	1 (0)	14 (0)
Family history of epilepsy in 1^st^/2^nd^ degree relatives, *n* (%)	50 (30.86)	5 (33.33)	5 (33.33)	1 (16.66)	1 (50)	1 (50)
Family history of febrile seizures in 1^st^/ 2^nd^ degree relatives, *n* (%)	9 (5.55)	2 (13.33)	0	6 (100)	0	0
Mild ID/borderline intellectual functioning, *n* (%)	10 (6.17)	6 (40)	1 (6.66)	1 (16.66)	2 (100)	0
Psychiatric comorbidities, *n* (%)	19 (11.73)	2 (13.33)	4 (26.66)	0	1 (50)	0
History of febrile seizures, *n* (%)	12 (7.41)	2 (13.33)	1 (6.66)	6 (100)	0	0
EEG focal spikes, *n* (%)	16 (9.88)	2 (13.33)	3 (20)	1 (16.66)	0	0
Photoparoxysmal response, *n* (%)	35 (21.60)	13 (86.66)	2 (13.33)	4 (66.66)	0	0

*EEG, electroencephalography; EMA, eyelid myoclonia with absences; GEFS+, generalized epilepsy with febrile seizures plus; GGE, genetic generalized epilepsy; ID, intellectual disability; IGE, idiopathic generalized epilepsy; SD, standard deviation*.

### paEEG Findings and GPFA/GPT Characteristics

The median time from epilepsy diagnosis to paEEG recording was 13 years (IQR 7–23).

SWDs and PWDs were recorded in 168 patients (83.3%) and were found exclusively during the sleep state in 22/168 subjects. The prevalence of SWD/PWD during wakefulness was frequent/abundant in 45 (22.2%), occasional/rare in 101 (50%), none in 56 (27.8%), whereas during sleep was frequent/abundant in 57 (28.1%), rare/occasional in 112 (55.2%) and none in 34 (16.7%).

The maximum SWD/PWD median frequency was 4 c/s (IQR 3–4) and the minimum median frequency was 3 c/s (IQR 3-−3). Focal epileptiform abnormalities were recorded in 23 patients (11.4%), focal slow waves in 10 (5%), and SWD/PWD asymmetry in 31 (15.3%).

**Figure 1:**
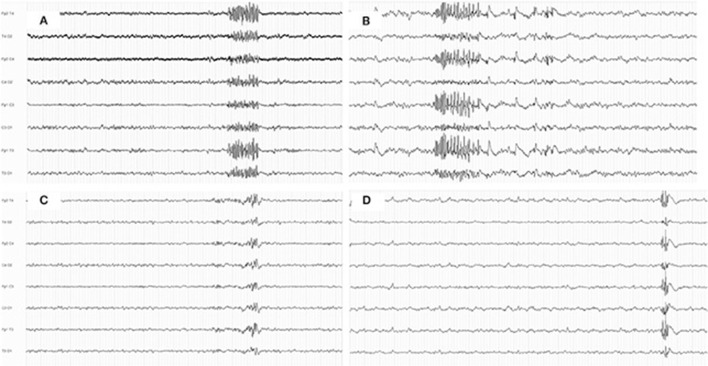
Examples of generalized fast discharges during sleep. **(A,B)** Examples of generalized paroxysmal fast activity in two patients **(A)** undefined genetic generalized epilepsy; **(B)** perioral myoclonia with absences; **(C,D)** examples of generalized polyspike train in two patients; **(C)** juvenile myoclonic epilepsy; **(D)** childhood absence epilepsy.

GPFA and/or GPT were found in 25 patients (12.4%) (Figure 1). GPFA only was found in 3/25 patients (12%) and GPT only was found in 1/25 patients (4%), while both GPFA and GPT were found in 21/25 patients (84%).

The mean frequency of generalized fast discharges was 13.5 c/s (SD 2.48) and the mean duration was 2.38 s (SD 1.06). GPFA/GPT was observed during the N2 stage of sleep in 24 patients (96%) and exclusively during the N1 stage in 1 patient. In 7/25 patients (28%), GPFA and/or GPT was found even during the waking state. EEG examples of GPFA and/or GPT are shown in Figure 1.

The generalized fast discharges recorded in the study population included 456 GPFA discharges and 444 GPT discharges. The median number of GPFA discharges among patients showing this EEG pattern was 12 (IQR 5–35, range 1-−52), whereas the median number of GPT discharges among those displaying this EEG pattern was 15 (IQR 6–19, range 3–149). SWD/PWD occurrence was observed in 14 patients (56%) after fast discharges. The total number of GPFA discharges was not significantly associated with the total number of GPT discharges (Spearman r = 0.31, *p* = 0.14), but it was strongly associated with GPFA duration (Spearman r = 0.69, *p* < 0.001).

### Electroclinical Characteristics of the Cohort Stratified by the Presence/Absence of Generalized Fast Discharges

**Figure 2:**
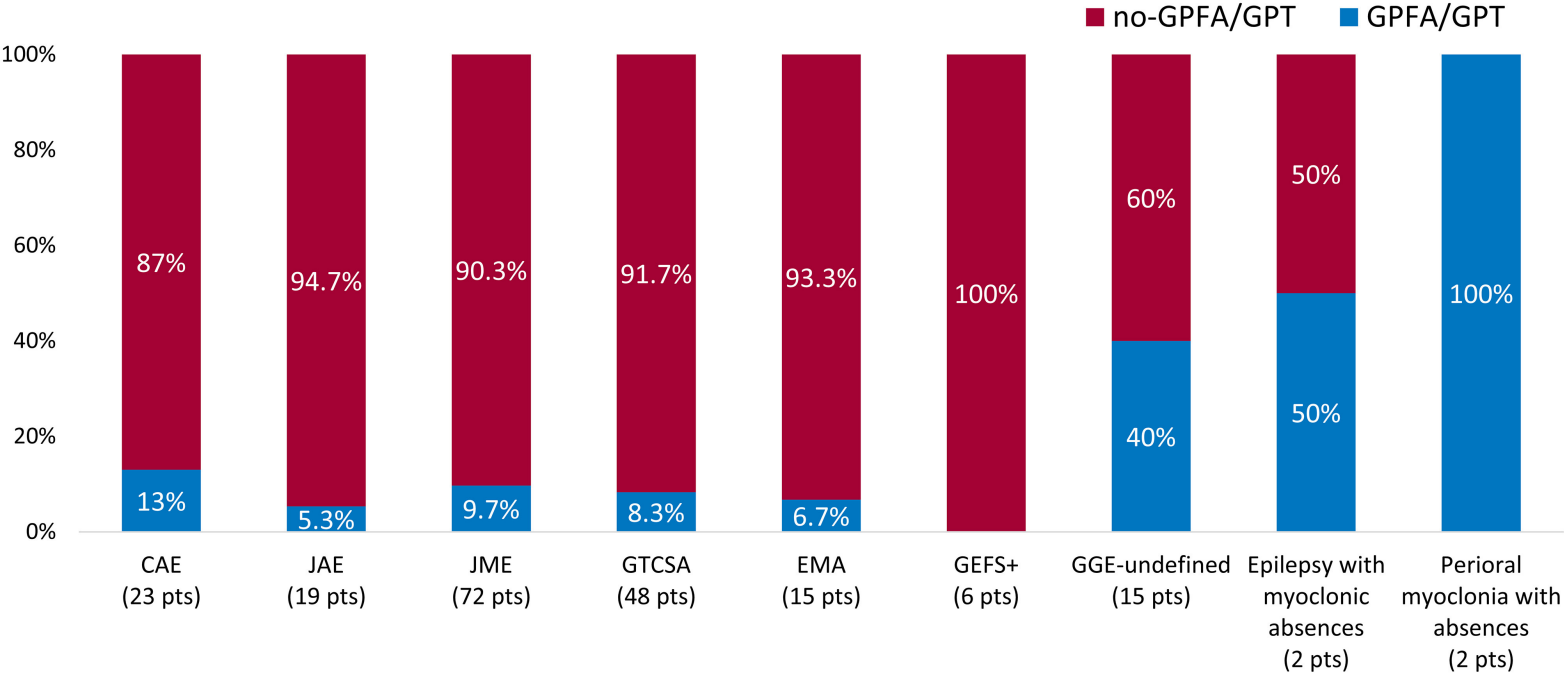
Generalized fast discharge distribution according to epilepsy syndrome. Proportion of patients showing EEG generalized fast discharges according to genetic generalized epilepsy (GGE) syndrome. The percentage of patients showing generalized fast discharges in each syndrome is reported in the blue bars. The absolute number of patients diagnosed with each syndrome is reported in brackets. GPFA, generalized paroxysmal fast activity; GPT, generalized polyspike train.

Time from epilepsy onset to paEEG recording was longer in patients with GPFA/GPT compared with those not showing these EEG patterns [20 years (IQR 8–32) vs. 12 years (IQR 6.5–21), *p* = 0.049], whereas the median time from paEEG to the last medical observation was similar between the two groups [GPFA/GPT: 8 years (IQR 4.5–10.5) vs. no-GPFA/GPT 7 years (IQR 4–13), *p* = 0.9], as well as the duration of paEEG recordings [GPFA/GPT 22.1 h (IQR 20.3–23.8) vs. no-GPFA/GPT 22.4 h (IQR 20.5–23.6), *p* = 0.9]. A significantly different GPFA/GPT distribution was observed between the analyzed GGE syndromes, being more frequent among patients with GGE syndromes other than IGE (10/40 GGE patients vs. 15/162 IGE patients, *p* = 0.007). The relative GPFA/GPT distribution according to specific GGE syndromes is represented in Figure 2. No significant differences were found according to seizure type experienced during epilepsy history or the presence of mild ID/borderline intellectual functioning. No patients with GPFA/GPT reported a personal or a family history of FS. A detailed description of electroclinical characteristics according to GPFA/GPT occurrence is presented in Table 2.

**Table 2:** Electroclinical characteristics of patients according to the presence or absence of GPFA/GPT during paEEG.

	**GPFA/GPT (+) (25 pts)**	**GPFA/GPT (–) (177 pts)**	***p* value**
Female sex, *n* (%)	15 (60)	99 (55.9)	0.8
Age at epilepsy onset, years, median (IQR)	13 (7.5–20)	13 (9–16.5)	0.8
Family history of epilepsy in 1^st^/2^nd^ degree relatives, *n* (%)	7 (28)	56 (31.6)	0.7
Family history of febrile seizures in 1^st^/2^nd^ degree relatives, *n* (%)	0	16 (9)	0.1
Mild ID/borderline intellectual functioning, *n* (%)	3 (12)	17 (9.6)	0.7
Psychiatric comorbidities, *n* (%)	4 (16)	22 (12.4)	0.5
History of febrile seizures, *n* (%)	0	21 (11.9)	0.08
Seizure types, *n* (%)			
Absence seizures, *n* (%)	15 (60)	83 (46.9)	0.2
Myoclonic seizures, *n* (%)	10 (40)	67 (37.9)	0.8
Generalized tonic-clonic seizures, n (%)	23 (92)	163 (92.1)	1
Eyelid myoclonia with absences, *n* (%)	4 (16)	20 (11.3)	0.5
EEG characteristics			
Background activity, Hz, median (IQR)	9 (8.5–10.5)	9.5 (9–11)	0.6
Focal epileptiform abnormalities, *n* (%)	5 (20)	17 (9.6)	0.16
Photoparoxysmal response, *n* (%)	3 (12)	51 (28.8)	0.09

*GPFA, generalized paroxysmal fast activity; GPT, generalized polyspike train; ID, intellectual disability; IQR, interquartile range; paEEG, 24-h prolonged ambulatory electroencephalography*.

### Correlations Between Generalized Fast Discharges and Outcome Measures

A total of 97 patients (48%) were seizure free during the 12 months preceding the paEEG, whereas absence seizures were reported in 50 patients (24.8%), myoclonic seizures in 27 (13.4%), and GTCS in 49 (24.3%). GPFA/GPT was less common in patients who experienced 12 months of freedom from all seizure types prior to paEEG [10/25 (40%) vs. 91/177 (51.4%), *p* = 0.3]. When focusing on different seizure types, GPFA/GPT occurrence was significantly associated with the persistence of absence seizures during the 12 months preceding paEEG recording [11/25 (44%) vs. 39/177 (22%), *p* = 0.02], whereas no significant differences were found in terms of other seizure types [myoclonic seizures: GPFA/GPT 4/25 (16%) vs. no GPFA/GPT 23/177 (13%), *p* = 0.7; GTCS: GPFA/GPT 8/25 (32%) vs. no GPFA/GPT 41/177 (23.2%), *p* = 0.3].

An early remission pattern of seizure control was significantly less frequent in patients with GPFA/GPT compared with those not exhibiting these EEG patterns (4 vs. 24.3%, *p* = 0.02).

Two-year seizure remission at the last medical observation was observed in 11/25 patients (44%) with GPFA/GPT and in 107/177 patients (60.5%) without GPFA/GPT (*p* = 0.1). Two-year seizure remission was found in 0/6 patients with GPFA/GPT during wakefulness, whereas these patients did not differ in terms of other electroclinical characteristics. The total number of GPFA discharges recorded during paEEG discriminated between patients with and without a 2-year seizure remission at the last medical observation (AUC = 0.77, 95% CI 0.57–0.97, *p* = 0.02) better than the number of GPT discharges (AUC = 0.65, 95% CI = 0.42–0.88, *p* = 0.2).

**Figure 3:**
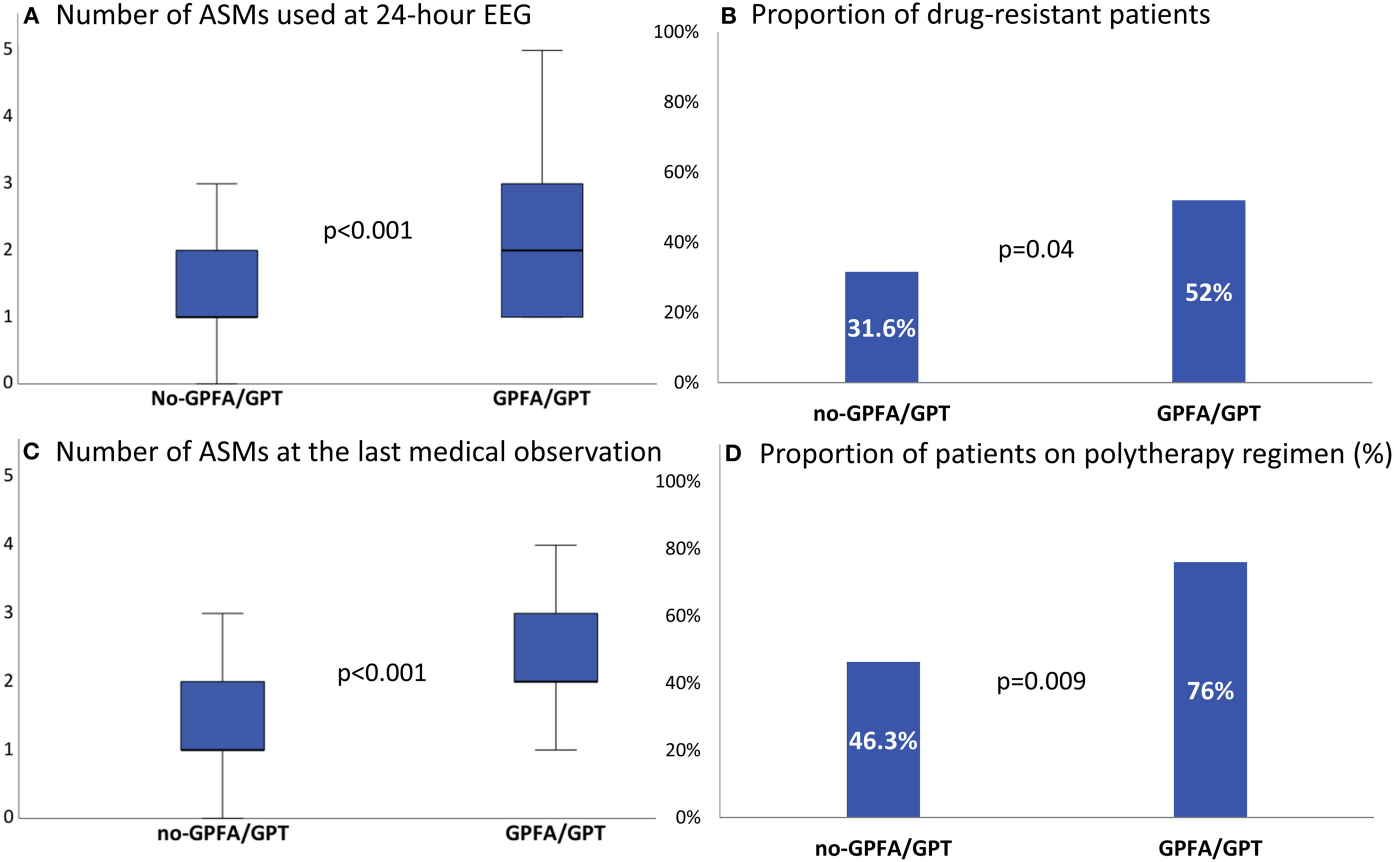
Antiseizure medication burden at different timepoints according to the presence or absence of generalized fast discharges. **(A,C)** Whisker plots representing the median number of antiseizure medications at different time points. **(B,D)** Bar graph showing the proportion of patients with drug-resistance and with a polytherapy regimen at the last medical observation. ASM, antiseizure medication; GPFA, generalized paroxysmal fast activity; GPT, generalized polyspike train.

The median number of ASMs at the time of paEEG was higher in patients with GPFA/GPT than in patients without these EEG patterns [2 (IQR 1–3) vs. 1 (IQR 1–2), *p* < 0.001], as was the median number of ASMs used at the last medical observation [2 (IQR 1.5–3) vs. 1 (IQR 1–2), *p* < 0.001] (Figure 3). Drug-resistant epilepsy was observed in 69/202 patients (34.2%) in the whole cohort, and it was found to be significantly more common in patients with GPFA/GPT during paEEG than in those without GPFA/GPT during paEEG [13/25 (52%) vs. 56/177 (31.6%), *p* = 0.04). A significantly higher number of patients with GPFA/GPT were on a polytherapy regimen (≥2 ASMs) at the last medical observation compared with patients without EEG fast discharges (76 vs. 46.3%, *p* = 0.009) (Figure 3). Multiple linear regression models confirmed the significant association between GPFA/GPT and the number of ASMs used at paEEG (beta = 0.27, *p* < 0.001, Supplementary Table 2) and at the last medical observation (beta=0.26, *p* < 0.001, Supplementary Table 3), whereas multivariable logistic regression models showed a significant association between GPFA/GPT and drug-resistant GGE (Supplementary Table 4).

Finally, GPFA/GPT (OR = 0.32, 95% CI = 0.11−0.92, *p* = 0.04), frequent/abundant SWD/PWD during sleep (OR = 0.21,95% CI = 0.07–0.61, *p* = 0.004) and a higher number of seizure types experienced during history (OR = 0.45, 95% CI = 0.28–0.74, *p* = 0.001) were the only factors independently associated with a reduced chance of achieving 2-year seizure remission at the last medical observation, according to multivariable logistic regression analysis (Table 3). The multivariable model showed an AUC of 0.73 (95% CI = 0.67–0.81, *p* < 0.001) for discriminating patients who achieved 2-year remission at the last medical observation from those who did not.

**Table 3:** Multivariable logistic regression analysis using 2-year remission at the last medical observation as a dependent variable.

**Variables**	**OR**	**95% CI**	***p* value**
Female sex (yes/not)	0.7	0.36–1.3	0.3
Age at epilepsy onset, years	1.02	0.97–1.07	0.4
Time from epilepsy onset to paEEG, years	1.01	0.98–1.04	0.4
Time from paEEG to the last medical observation, years	1.06	0.99–1.12	0.054
History of febrile seizures (yes/not)	0.41	0.14–1.15	0.09
Psychiatric comorbidities (yes/not)	0.63	0.24–1.64	0.3
History of photosensitivity (yes/not)	0.56	0.27–1.14	0.1
Number of seizure types ever experienced, n	0.45	0.28–0.74	0.001*
SWD/PWD prevalence during sleep			
None (reference category) (yes/not)			
Rare/occasional (yes/not)	0.39	0.14–1.1	0.08
Frequent/abundant (yes/not)	0.21	0.07–0.61	0.004*
GPFA/GPT (yes/not)	0.32	0.11–0.92	0.04*

*CI, confidence interval; GPFA, generalized paroxysmal fast activity; GPT, generalized polyspike train; paEEG, prolonged ambulatory EEG recording; OR, odd ratio; PWD, polyspike-wave discharge; SWD, spike-wave discharge. The asterisks indicate statistically significant variables (p < 0.05)*.

## Discussion

To the best of our knowledge, this is the first study in which paEEGs were systematically reviewed to investigate the presence of EEG fast discharges in a large cohort of GGE patients thoroughly characterized in accordance with the latest classification proposals. The careful revision of EEG tracings allowed us to identify GPFA/GPT in 12.3% of our cohort, a higher proportion compared with previous literature findings (18, 19). This discrepancy might be related, on the one hand, to the systematic review of paEEGs in all patients, and on the other, to the inclusion of the entire spectrum of GGE.

After a careful classification of our patients according to well-defined GGE syndromes, we observed that GPFA/GPT could be documented in both typical IGE and in other GGE syndromes. However, when focusing on the relative distribution of GPFA/GPT, it appeared significantly more frequent in syndromes other than IGE (25 vs. 9.7%), namely undefined-GGE, eyelid myoclonia with absences, epilepsy with myoclonic absences and perioral myoclonia with absences, which have been previously associated with a less benign course (11, 12, 20). By documenting that GPFA/GPT could represent a common EEG pattern in the generalized epilepsies outside the IGE umbrella, our study also contributes to enrich the characterization of these poorly defined GGE syndromes. Indeed, the presence of GPFA/GPT further supports the hypothesis that these syndromes could be considered as “intermediate” forms of generalized epilepsies, placed halfway between IGEs and epileptic/developmental encephalopathies (9, 21, 22).

From an electro-clinical perspective (i.e., beyond syndromic classification), we found that GPFA/GPT was not strictly associated with specific clinical features and could be observed independently of mild ID/borderline intellectual functioning, specific EEG findings, or seizure types experienced during history. Conversely, GPFAs/GPTs were associated with the persistence of absence seizures at the year of paEEG, in accordance with previous findings originally describing GPFAs in patients with absences persisting in adult life (23).

In our study we also explored several outcome measures in an attempt to unravel the prognostic significance of generalized fast discharges in GGE patients. According to our data, patients with GPFA/GPT had lower rates of early remission and a higher drug load during history, at the time of paEEG, and at the last medical observation. Interestingly, we also found a prognostic impact of a higher number of GPFA discharges recorded during paEEG, which was found to discriminate between patients who achieved remission at the last medical observation and those who did not. These findings were corroborated by our multivariable analysis, which confirmed GPFA/GPT as a significant independent predictor of both drug-resistant GGE and of a reduced chance of achieving 2-year seizure remission at the last medical observation. Overall, our data seem to support that EEG fast discharges represent an EEG biomarker of more difficult-to-treat GGE (6, 24, 25). However, the lack of systematic paEEG use at epilepsy onset prevented us from understanding whether generalized fast discharges represent an endophenotypic trait of more refractory GGE from its very onset or an epiphenomenon of prolonged unremitting seizures during the epilepsy course. Indeed, previous longitudinal studies have shown that unremitting seizure activity may determine a progressive impairment of GABA-mediated inhibition (26, 27), which has been proposed as a major pathophysiological determinant of generalized fast discharge occurrence (28).

Regarding electrophysiological characteristics, the described EEG patterns were analyzed in terms of morphology and frequency and in relation to physiological state. The prominent occurrence of GPFA during sleep (especially during the N2 stage) was confirmed in our cohort (2, 24). Previous authors have hypothesized that NREM sleep could represent a permissive neural environment for the production of GPFA, possibly in relation to the prominent occurrence of slow oscillations during this physiological state, which have been demonstrated to help facilitate fast activity bursts both in animal models and in LGS patients (29–31). The co-existence of GPFA and GPT in the majority of patients (84%) and the absence of EEG differences other than discharge duration between GPFA and GPT seem to support a shared pathophysiological mechanism between GPFA and GPT (6). In addition, none of the subjects with GPFA/GPT during wakefulness achieved remission at the last observation, suggesting a more negative prognostic impact of these EEG patterns when found in this physiological state.

Although our study provided interesting new findings, it was limited by several factors, including: (1) the retrospective nature of the study with its intrinsic bias; (2) the enrolment of patients followed in tertiary epilepsy care centers, where higher rates of drug resistance may be found; (3) the low proportion of paEEG performed at epilepsy onset, early in the clinical history, and in untreated patients (the study only enrolled a few naïve cases); and (4) the relatively small sample size, which determined a low representation of rare GGE syndromes, such as epilepsy with myoclonic absences and perioral myoclonia with absences; (5) the possible recruitment bias related to the inclusion of patients with at least one paEEG recording, which may have determined an overrepresentation of more refractory epilepsy cases.

Future studies addressing the topic of generalized fast discharges in GGE patients could contribute to the understanding of the prognostic significance of GPFA/GPT by prospectively performing paEEG in treatment-naïve patients at epilepsy onset. In addition, hypothesis-driven genetic and neuroimaging studies could help elucidate the biological and pathophysiological background of GPFA/GPT by comparing patients who exhibit these discharges with those who do not.

In conclusion, our study shows that GPFA/GPT is more common than expected in GGE when considering the entire GGE spectrum. We highlighted that these EEG patterns may be found along the entire GGE continuum, though their detection was more frequent in less benign GGE phenotypes. Finally, we confirmed that EEG fast discharges were associated with difficult-to-treat GGE, as evidenced by the multivariable model and the higher drug burden in patients with GPFA/GPT.

## Data Availability Statement

The raw data supporting the conclusions of this article will be made available by the authors, without undue reservation.

## Ethics Statement

The studies involving human participants were reviewed and approved by Policlinico Umberto I Ethical Committee. The patients/participants provided their written informed consent to participate in this study.

## Author Contributions

ECI, FAB, and CDB contributed to conception, design of the study, and wrote the first draft of the manuscript. ECI performed the statistical analysis. LM, AMo, and FI wrote sections of the manuscript. ECI, LM, AMo, BO, EMS, AMa, MF, JF, FP, ATG, and FI had a major role in data acquisition. All authors contributed to manuscript revision, read, and approved the submitted version.

## Funding

This project has received funding from the European Union's Horizon 2020 research and innovation programme under grant agreement no. 952455.

## Conflict of Interest

The authors declare that the research was conducted in the absence of any commercial or financial relationships that could be construed as a potential conflict of interest.

## Publisher's Note

All claims expressed in this article are solely those of the authors and do not necessarily represent those of their affiliated organizations, or those of the publisher, the editors and the reviewers. Any product that may be evaluated in this article, or claim that may be made by its manufacturer, is not guaranteed or endorsed by the publisher.
