# Supplementary material for: Generalized Fast Discharges Along the Genetic Generalized Epilepsy Spectrum: Clinical and Prognostic Significance
Source: Front Neurol. 2022 Mar 10;13:844674. doi: 10.3389/fneur.2022.844674 (PMC8960043; doi:10.3389/fneur.2022.844674)
Supplement: Supplementary file 1 [file Data_Sheet_1.docx]

| **Supplementary table 1. Main electroclinical characteristics stratified by idiopathic generalized epilepsy syndrome** | | | | |
| --- | --- | --- | --- | --- |
|  | CAE  (23 pts) | JAE  (19 pts) | JME  (72 pts) | GTCSA  (48 pts) |
| Female sex, n (%) | 11 (47.8) | 12 (63.2) | 46 (63.9) | 22 (45.8) |
| Age at epilepsy onset, years, mean (SD) |  |  |  |  |
| Family history of epilepsy in 1^st^/ 2^nd^ degree relatives, n (%) | 10 (43.5) | 6 (31.6) | 24 (33.3) | 10 (20.8) |
| Family history of febrile seizures in 1^st^/ 2^nd^ degree relatives, n (%) | 2 (8.7) | 2 (10.5) | 5 (6.9) | 0 |
| Borderline intellectual functioning, n (%) | 1 (4.3) | 2 (10.5) | 4 (5.6) | 3 (6.3) |
| Psychiatric comorbidities, n (%) | 3 (13) | 3 (15.8) | 11 (15.3) | 2 (4.2) |
| History of febrile seizures, n (%) | 2 (8.7) | 1 (5.3) | 5 (6.9) | 4 (8.3) |
| EEG focal spikes, n (%) | 2 (8.7) | 1 (5.3) | 7 (9.7) | 5 (10.4) |
| Photoparoxysmal response, n (%) | 3 (13) | 2 (10.5) | 22 (30.6) | 8 (16.7) |
| Abbreviations: CAE = childhood absence epilepsy; EEG = electroencephalography; GTCSA = idiopathic generalized epilepsy with generalized tonic-clonic seizure alone; JAE = juvenile absence epilepsy; JME = juvenile myoclonic epilepsy; SD = standard deviation | | | | |

| **Supplementary table 2. Linear regression model using as dependent variable the number of antiseizure medications used at the moment of paEEG recording** | | |
| --- | --- | --- |
| Variables | Beta (95% CI) | p value |
| Age at epilepsy onset, years | -0.06 (-0.2-0.08) | 0.4 |
| Time from epilepsy onset to paEEG recording, years | 0.35 (0.22-0.49) | <0.001* |
| Female sex (yes/not) | -0.07 (-0.2-0.05) | 0.3 |
| Psychiatric comorbidities (yes/not) | 0.06 (-0.06-0.19) | 0.4 |
| Number of seizure types ever experienced, n | 0.1 (-0.03-0.24) | 0.1 |
| Frequent/abundant SWD/PWD during sleep (yes/not) | 0.09 (-0.05-0.21) | 0.1 |
| GPFA/GPT (yes/not) | 0.27 (0.14-0.39) | <0.001* |
| Abbreviations: GPFA = generalized paroxysmal fast activity ; GPT = generalized polyspike train ; paEEG = prolonged ambulatory EEG ; PWD = polyspike-wave discharge ; SWD = spike-wave discharge. The asterisks indicate statistically significant variables (p<0.05) | | |

| **Supplementary table 3. Linear regression model using as dependent variable the number of antiseizure medications used at the last medical observation** | | |
| --- | --- | --- |
| Variables | Beta (95% CI) | p value |
| Age at epilepsy onset, years | -0.03 (-0.18-0.1) | 0.6 |
| Time from the epilepsy onset to the last follow-up visit, years | 0.27 (0.13-0.4) | <0.001* |
| Female sex (yes/not) | 0.06 (-0.06-0.19) | 0.3 |
| Number of seizure types ever experienced, n | 0.12 (-0.01-0.26) | 0.08 |
| Psychiatric comorbidities (yes/not) | 0.09 (-0.04-0.22) | 0.2 |
| EEG focal spikes (yes/not) | 0.06 (-0.06-0.18) | 0.4 |
| GPFA/GPT (yes/not) | 0.26 (0.15-0.38) | p<0.001* |
| Abbreviations: GPFA = generalized paroxysmal fast activity ; GPT = generalized polyspike train. The asterisks indicate statistically significant variables (p<0.05) | | |

| **Supplementary table 4. Multivariable logistic regression model using drug-resistance as dependent variable** | | |
| --- | --- | --- |
| Variables | Odds Ratio (95% CI) | p value |
| Age at epilepsy onset, years | 0.98 (0.94-1.04) | 0.6 |
| Time from epilepsy onset to the last medical observation, years | 1.01 (0.97-1.03) | 0.9 |
| Female sex (yes/not) | 1.78 (0.91-3.46) | 0.1 |
| History of febrile seizures (yes/not) | 1.83 (0.66-5.1) | 0.2 |
| Psychiatric comorbidities (yes/not) | 1.28 (0.51-3.21) | 0.6 |
| Number of seizure types ever experienced, n | 2.01 (1.26-3.2) | 0.004* |
| Photosensitivity (yes/not) | 1.59 (0.78-3.23) | 0.2 |
| SWD/PWD prevalence during sleep |  |  |
| None (reference category) (yes/not) |  |  |
| Rare/occasional (yes/not) | 1.75 (0.63-4.85) | 0.3 |
| Frequent/abundant (yes/not) | 3.21 (1.15-8.7) | 0.03* |
| GPFA/GPT (yes/not) | 2.93 (1.06-8.08) | 0.04* |
| Abbreviations: GPFA = generalized paroxysmal fast activity ; GPT = generalized polyspike train ; PWD = polyspike-wave discharge ; SWD = spike-wave discharge . The asterisks indicate statistically significant variables (p<0.05) | | |
