# Supplementary material for: Generalized Fast Discharges Along the Genetic Generalized Epilepsy Spectrum: Clinical and Prognostic Significance
Source: Front Neurol. 2022 Mar 10;13:844674. doi: 10.3389/fneur.2022.844674 (PMC8960043; doi:10.3389/fneur.2022.844674)
Supplement: Supplementary file 2 [file Data_Sheet_2.pdf]

Generalized fast discharges along the genetic generalized epilepsy spectrum: clinical and prognostic significance

Emanuele Cerulli Irelli,[1] Francesca A Barone, [1] Luisa Mari,[2] Biagio Orlando,[1] Enrico M Salamone,[1] Angela Marchi,[2] Alessandra Morano,[1] Martina Fanella,[1] Jinane Fattouch,[1] Fabio Placidi,[2] Anna T Giallonardo,[1] Francesca Izzi,[2] Carlo Di Bonaventura[1]

The four idiopathic generalized epilepsy syndromes (namely childhood absence epilepsy, juvenile absence epilepsy, juvenile myoclonic epilepsy, idiopathic generalized epilepsy with generalized tonic-clonic seizures alone) were diagnosed based on the latest classification proposal.[1]

Eyelid myoclonia with absences was diagnosed according to the following inclusion and exclusion criteria:[2] 1) age at onset between 2 and 14 years; 2) history of eyelid myoclonia with or without absences; 3) history of photosensitivity and/or eye closure sensitivity; 4) EEG generalized spike-wave discharges (SWDs) and/or polyspike-wave discharges (PWDs); 5) normal neuroimaging (when available) and neurological examination. We excluded patients with 1) cognitive deficits other than borderline intellectual functioning and mild ID; and 2) myoclonic jerks in body parts other than the eyelids.

Epilepsy with myoclonic absences was diagnosed according to the following inclusion and exclusion criteria:[3] 1) Age at onset within 1 year and 12 years; 2) history of myoclonic absences, characterized by different degrees of awareness impairment, associated with an increased tonic contraction of different body parts (typically of the upper limbs) with superimposed myoclonic jerks; 2) EEG SWDs and/or PWDs; 3) normal neuroimaging and neurological examination. Patients with cognitive deficits other than borderline intellectual functioning and mild ID were excluded.

Perioral myoclonia with absences was diagnosed according to the following inclusion and exclusion criteria:[4] 1) late childhood or adolescent onset of epilepsy; 2) history of absences associated with variable degrees of awareness impairment, associated with orbicularis oris muscle myoclonic jerks; 3) EEG SWDs and/or PWDs; 4) normal neuroimaging and neurological examination. Patients with cognitive deficits other than borderline intellectual functioning and mild ID were excluded.

Generalized epilepsy with febrile seizures plus was diagnosed according to the following inclusion and exclusion criteria 1) history of both febrile seizures (FS) and generalized afebrile seizures, together with a family history of FS in 1st or 2nd-degree relatives; 2) EEG SWDs and/or PWDs; 3) normal neuroimaging and neurological examination. Patients with cognitive deficits other than borderline intellectual functioning and mild ID were excluded.

Patients who had genetic generalized epilepsy (generalized seizure types, SWDs and/or PWDs, normal neuroimaging, normal intellectual functioning/borderline intellectual functioning) but did not fit neatly into a recognized epilepsy syndrome, have been classified as genetic generalized epilepsy without a specific epilepsy syndrome.[1]

References

1. Hirsh E, French J, Sheffer IE, et al. ILAE Definition of the Idiopathic Generalized Epilepsy Syndromes: Position Statement by the ILAE Task Force on Nosology and Definitions (2021) Available at: https://www.ilae.org/guidelines/definition-and-classification/proposed-classification-and-definition-of-epilepsy-syndromes/proposed-classification-idiopathic-generalized-epilepsies

2. Genton P, Bureau M. Epilepsy with myoclonic absences. CNS Drugs. 2006;20:911-916.

3. Striano S, Striano P, Nocerino C, et al. Eyelid myoclonia with absences: an overlooked epileptic syndrome? Neurophysiol Clin. 2002;32:287-296

4. Bilgiç B, Baykan B, Gürses C, Gökyiğit A. Perioral myoclonia with absence seizures: a rare epileptic syndrome. Epileptic Disord. 2001;3:23-27.
