# Supplementary material for: Generalized Fast Discharges Along the Genetic Generalized Epilepsy Spectrum: Clinical and Prognostic Significance
Source: Front Neurol. 2022 Mar 10;13:844674. doi: 10.3389/fneur.2022.844674 (PMC8960043; doi:10.3389/fneur.2022.844674)
Supplement: Supplementary file 3 [file Presentation_1.PPTX]

## Slide 1
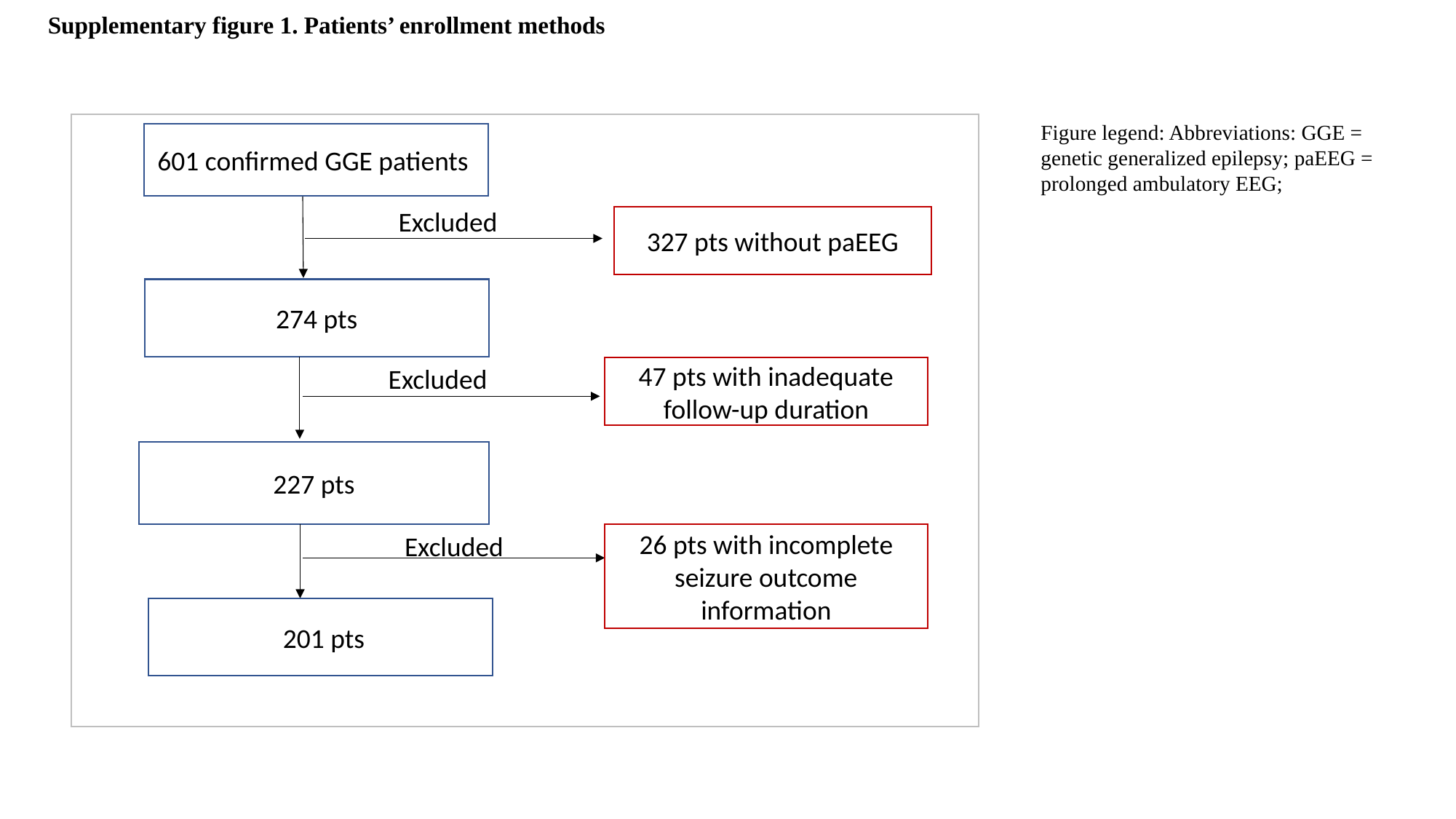

Supplementary figure 1. Patients’ enrollment methods
Figure legend: Abbreviations: GGE = genetic generalized epilepsy; paEEG = prolonged ambulatory EEG;
601 confirmed GGE patients
Excluded
327 pts without paEEG
274 pts
Excluded
47 pts with inadequate follow-up duration
227 pts
Excluded
26 pts with incomplete seizure outcome information
 201 pts
